# Moving beyond discrete categories in motor cognition

**DOI:** 10.3758/s13423-026-02950-3

**Published:** 2026-07-07

**Authors:** Roland Pfister, Greg Huffman, James R. Brockmole

**Affiliations:** 1 Experimental Psychology, Trier University, Trier, Germany; 2 Institute of Cognitive and Affective Neuroscience, Trier University, Trier, Germany; 3 Scoring Technologies, St. George, UT USA; 4 Department of Psychology, University of Notre Dame, Notre Dame, IN USA

**Keywords:** Perception and action, Motor control, Feature binding and retrieval

## Abstract

Theories on human action control assume the human mind copes with the computational challenge of selecting and performing goal-directed movements by recycling previous action plans. Feature binding assists this recycling by integrating stimulus and response features into episodic representations. Perhaps surprisingly, however, previous evidence for binding and retrieval is confined to categorical perceptual and motor features that define coarse-grained decisions. Here we show that such episodic representations can also incorporate continuous, metric information. These conclusions emerge from two experiments in which participants performed whole-arm pointing movements to varying target locations with their left or right hand. Short inter-trial distances between successive locations benefitted response repetitions (i.e., responding with the same hand in consecutive trials) whereas increasing distances benefitted response changes. This effect emerged as a direct function of metric distance. Feature binding and retrieval thus seem to go beyond categorical decision making, either by including metric information about current stimulation or by providing direct access to an agent’s motor repertoire.

## Introduction

Action planning requires specifying what effector to move in what way to achieve a desired goal. This process is effortful, so the human cognitive system comes with an elegant mechanism to recycle previously compiled action plans when re-encountering a situation. This mechanism is often called *binding* as it bundles perceptual and motor information into integrated representations (Frings et al., 2020, 2024; Hommel et al., 2001; Kahneman et al., 1992). These bindings can persist even after action execution so that action plans can be *retrieved* swiftly upon re-encountering a situation that resembles the perceptual information contained in available bindings. Such retrieval supports flexible and efficient action control by providing a short-cut to appropriate and adaptive behavior.

Evidence in favor of binding and retrieval appears highly convincing at first sight (Frings et al., 2020; Henson et al., 2014). Studies in this research tradition have shown that re-encountering previous stimulation speeds up responses that had been performed in the presence of this stimulation (Hilchey et al., 2018; Huffman et al., 2020; Kahneman et al., 1992; Quinlan, 1998). This is particularly apparent in controlled response-time tasks such as the distractor-response binding paradigm (Frings et al., 2007). Here, participants identify different possible target stimuli placed within a set of distractors. Responses are made via keypresses in order to associate each potential target with a different action. Binding and retrieval of action plans become apparent in analyses of response times across successive trials: Typically, trials in which target and response repeat are faster than response changes. Critically, this facilitation is increased when distractors also repeat across trials and, conversely, it is diminished if distractors change. In the first case, the target and the distractor together correctly activate a previous action, hastening its implementation. In the second case, however, the distractors errantly activate an incompatible action, creating interference that takes time to resolve (for related findings, see, e.g., Bogon et al., 2017; Frings & Rothermund, 2011; Hollingworth, 2007; Neill, 1997).

Closer inspection of the available evidence reveals a striking limitation of previous work, however, as it has almost exclusively relied on *categorical* distinctions between possible stimulus features (e.g., clear color categories such as red and green) and possible response features (e.g., binary response choices such as left and right button presses). What studies have thus probed for is whether the binary decision of performing a left versus right response was bound to categorical features of incoming stimulation and retrieved when re-encountering that *exact* situation again. These methodological choices appear reasonable, because they allow for elegant experiments with orthogonal designs. However, they also pave the way for an entirely different reading of the literature: Rather than assuming binding and retrieval of sensory information and action plans, previous evidence can be explained alternatively in terms of categorical decision-making (see also Foerster et al., 2026). Whereas the canonical interpretation of binding and retrieval effects implies that perceptual codes retrieve actual action plans, including corresponding motor specifications, the observed response-time effects could alternatively reflect a speed-up of the decision for a certain alternative irrespective of the actions that follow from this decision. Conversely, retrieval might not be based on perceptual information but rather on the categorization performed on perceptual input. Therefore, it is currently unclear whether the prolific field of research on human action control actually taps into perceptual and motor representations, or whether it is confined to categorical decision-making instead. Generalizing the available evidence to actual perception and action planning would require the observation of binding and retrieval effects for non-categorical, continuous features as well, because each stimulus will come with non-categorical information and every action plan will eventually require precise parameters of the motor movement to be specified (Butterfill & Sinigaglia, 2014). That is, each action eventually has to extend from a categorical decision (e.g., “press the left button”; “ring the doorbell”) to specify precise motor parameters of the required muscle impulses that orchestrate an overt movement. This is precisely what the present experiments set out to explore by studying binding and retrieval for continuous stimulus and response locations.

Initial work on this question has capitalized on the observation that even simple keypress responses in a binary choice response task come with several relevant continuous response features. These studies, therefore, aimed to gather evidence for binding and retrieval of continuous features of simple keypress responses, such as their duration (i.e., the time from keypress to key release; Pfister et al., 2023), or the force exerted during action execution (Varga et al., 2024). The clear prediction of these studies was that retrieving an action plan (e.g., by re-encountering previous stimulation) should render response durations and force profiles similar to what had been performed when binding perceptual and motor features together. Perhaps surprisingly, however, binding and retrieval effects on these variables were small at best, suggesting that binding largely operates on categorical representations rather than on the information that is required to actually control a body movement (Bogon et al., 2023; Mocke et al., 2022; Pfister et al., 2022; Varga et al., 2022, 2024; Zwickel et al., 2010). Another reading of these findings, however, is that the chosen variables depend heavily on real-time control of an action after it is initiated rather than action planning per se (Woodworth, 1899). If so, response durations and force profiles would be poorly related to the binding and retrieval mechanisms that likely operate during action planning and initiation (Horváth et al., 2018).

Instead of sampling subtle continuous features in categorical keypress tasks, the present experiments targeted extended movements to elucidate whether binding and retrieval would also incorporate actual, continuous features for these movements. To this end, we had participants perform whole-arm pointing movements towards visual stimuli on a touchscreen monitor and asked whether the exact, metric location of the pointing target would become bound and retrieved like categorical features in previous approaches.

## Experiment 1

### Methods

#### Open science practices

We report all manipulations, all measures in the study, and all data exclusions. We pre-registered sample size, procedure, and analyses (https://aspredicted.org/sc3m-88ww.pdf). Anonymized raw data and analysis scripts are available on the Open Science Framework (https://osf.io/f2n4s/).

#### Participants

Thirty-four participants from the University of Notre Dame participated in exchange for course credit. All participants reported normal or corrected-to-normal visual acuity and color vision. We determined the sample size based on an a priori power analysis to achieve a power of 1-β ≥ 80% assuming at least medium-sized effects of Cohen’s *d*_*z*_ = 0.5. Because binding and retrieval effects for categorical features have often been reported in the range of *d*_*z*_ > 1.0 (e.g., Frings et al., 2007; Janczyk et al., 2023), actual power likely exceeds 80% by a substantial margin (1-β ≥ 99% for *d*_*z*_ = 1.0).

#### Stimuli and apparatus

Participants completed the experiment using a PC connected to an LCD touchscreen monitor (screen resolution: 1,920 × 1,080; refresh rate: 60 Hz). The experiment was programmed using Matlab (MathWorks, Natick, MA, USA) with the Psychophysics toolbox. The fixation was a black [RGB: (000,000,000)] circle subtending 0.2° visual angle. The target stimulus was a blue [RGB: (000,000,200)] or green [RGB: (000,200,000)] circle subtending 2° visual angle. Stimuli were presented on a gray background [RGB: (200,200,200)]. Participants responded using a standard US-style QWERTY keyboard and the touchscreen monitor.

#### Procedure

**Fig. 1:**
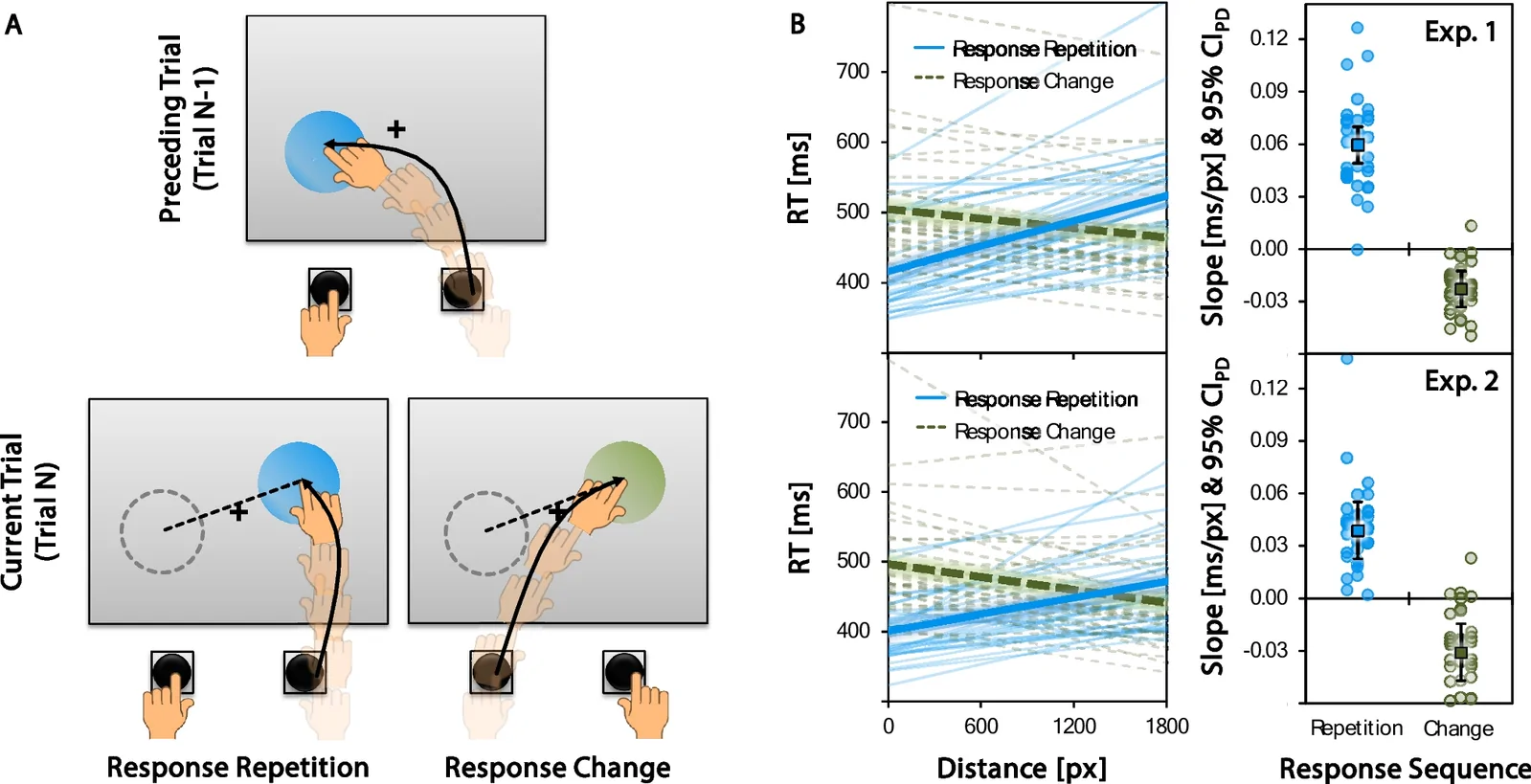
Design and results of the experiments. **(A)** To probe for binding and retrieval of continuous stimulus and response information, we asked participants to reach for a colored stimulus on a touchscreen. Stimulus color (blue vs. green) instructed the correct response hand, and response sequence (repetition vs. change) varied across trials. We analyzed performance as a function of response sequence and target-target distance across trials (dashed line). Binding and retrieval of continuous features would be evident in an interaction of response sequence and distance. In Experiment 1, stimulus location repeated in 50% of the trials and changed to a random other coordinate in the other 50%. Experiment 2 replicated the design with fully random locations across trials. **(B)** *Left plots:* Reaction time (RT) as a function of response sequence and target-target distance for Experiment 1 (top) and Experiment 2 (bottom). Thick lines represent group means while thin lines represent individual participants. *Right plots:* Mean and individual slopes of the distance effects in both conditions. Positive slopes indicate increasing RTs with increasing distance

Figure 1A shows a summary of the experimental design and procedure. Each trial began with a centrally presented fixation dot. Participants were asked to fixate on that location, but no eye tracking was employed to confirm compliance. Whenever only the fixation was on screen, participants were instructed to hold down the “Z” and “/?” keys using their left and right index fingers so that both hands were located on the same horizontal row of keys. After holding down both keys for 500 ms, a green or blue target appeared, either at the same location as the previous target or at a randomly determined different location (on the first trial the target always appeared at a random location). Half of the trials were exact location repetitions whereas the other half of the trials featured randomly varying positions as compared to the preceding trial. Participants were instructed to release one of the keys and touch the target stimulus with their left or right index finger. The correct response on a given trial was determined by the color of the target stimulus. Color-response mappings were counterbalanced across participants. Participants were asked to respond as quickly as they could without sacrificing accuracy. If they released a key before the target appeared, responded using the wrong hand, or missed the target on the screen, the trial was counted as an error and feedback was given to the participant.

#### Design

The experiment was a full within-subjects design with two factors. The first factor was *location sequence*. On one half of trials, the target appeared at precisely the same location as in the preceding trial (location repetition; distance = 0 px), whereas target location was randomly determined on the other half of trials (location change; distance > 0 px). These trial types appeared in random order. The second factor was *response sequence* (response repetition vs. change) so that the color that indicated the correct response hand in the current trial either repeated the previous color or changed to the alternative color. Half the trials required a left-hand response and the other half required a right-hand response, with color-hand mapping counterbalanced across participants. Each combination of location sequence and color was repeated 100 times each for a total of 400 trials.

#### Hypotheses

Our main interest was the question of whether binding and retrieval include non-categorical, continuous features. As a continuous feature we varied the spatial location of the target stimulus across trials. A simple, categorical integration of the spatial location would predict a categorical distinction between location repetition and location change trials. Full integration of the continuous, metric location would instead predict a pronounced role of the exact inter-trial distance between two successive locations, i.e., pronounced differences even within location-change trials. We thus tested how the inter-trial distance between two successive locations promoted either response repetitions (repeated pointing with the same hand) or response changes.

#### Analytical plan

Our main analysis employed linear mixed-effects modeling for reaction times (i.e., the time from stimulus onset to key release) and corresponding generalized linear mixed-effects models for error data (considering hand errors only). Response sequence (repetition vs. change) was modeled as a fixed effect in all models (coded as −0.5 for repetitions and +0.5 for changes), and subject was included as a random effect. Target-target distance was modeled either as a continuous predictor (coding the Euclidean distance in pixels) or as a binary, categorical predictor for comparison (location repetition vs. location change). In the continuous case, we centered the predictor variable separately for each participant and scaled it to a value range comparable to the first predictor (scaled distance = distance/1,000). All models were fit via the lme4 package version 1.1–23 in R (Bates et al., 2015).

### Results

#### Data exclusions

Trials in which participants responded too soon were removed prior to analysis (0.8%). We then screened the data for errors and excluded trials in which participants missed the target (1.2%). Trials in which participants responded with the wrong hand (3.8%) were retained for error analysis but excluded for the analysis of reaction times. We also removed the first trial of each participant to allow for meaningful sequential analyses. Finally, we screened the preprocessed data for outlier reaction times and excluded trials with reaction times deviating more than 2.5 standard deviations from the participant’s mean reaction time (2.1%; for reaction-time analysis only). As per our pre-registration, we discarded the data of one participant entirely due to having only 137/400 valid trials for analyses.

#### Reaction times

Figure 1B shows reaction times as a function of response sequence and target-target distance (upper panel). We fit and compared multiple models to determine which model best accounted for the data, using random-intercept models because full random-slopes models produced singular fits for selected analyses (all results replicate when fitting with random slopes but correlations between random effects removed). To gather first evidence for binding and retrieval of continuous features, we modeled distance as a continuous, metric predictor and fit a saturated model including the interaction of distance and response sequence. This model suggested longer reaction times for response changes than for response repetitions, *β* = 58.97 (SE = 1.94) and longer reaction times with increasing distance, *β* = 60.37 (SE = 3.02). Crucially, a strong interaction indicated that response repetitions were only faster than response changes at short distances whereas a tendency for a reversed effect was visible at long distances, *β* = −82.37 (SE = 4.28). To follow up on this observation we compared the saturated model to a main-effects model excluding the interaction. This model comparison clearly favored the saturated model, χ^2^(1) = 364.66, *p* <.001. A somewhat similar pattern emerged when coding distance as a binary predictor. Again, the saturated model included two sizeable main effects (response sequence: *β* = 59.39, *SE* = 1.96; distance: *β* = 40.52, *SE* = 2.72) that were qualified by an interaction, *β* = −58.07 (*SE* = 3.91), and a comparison to a corresponding main effects model favored the saturated model including the interaction, χ^2^(1) = 179.05, *p* <.001.

The reliable interaction of response sequence and distance supports the idea that the target and response location became integrated in an action plan and retrieved this action plan upon re-encountering a stimulus at a sufficiently similar location. These results, however, do not allow for deciding whether location was indeed represented in a continuous way or whether participants might have formed a categorical representation of “same location” versus “different location” instead. To determine whether location was best accounted for as a categorical predictor or as a continuous predictor, we computed the relative likelihood ℒ_relative_ of the more complex continuous model compared to the categorical model (e.g., Azzalini, 1996). We opted to compute this statistic instead of the above model comparisons because ℒ_relative_ can accommodate non-nested models. It thus allows for deciding between alternative models based solely on the model’s information loss as computed via Akaike’s Information Criterion (AIC):

1 $${\mathcal{L}}_{relative}= {e}^{\frac{{AIC}_{continuous}-{AIC}_{categorical}}{2}}$$

**Fig. 2:**
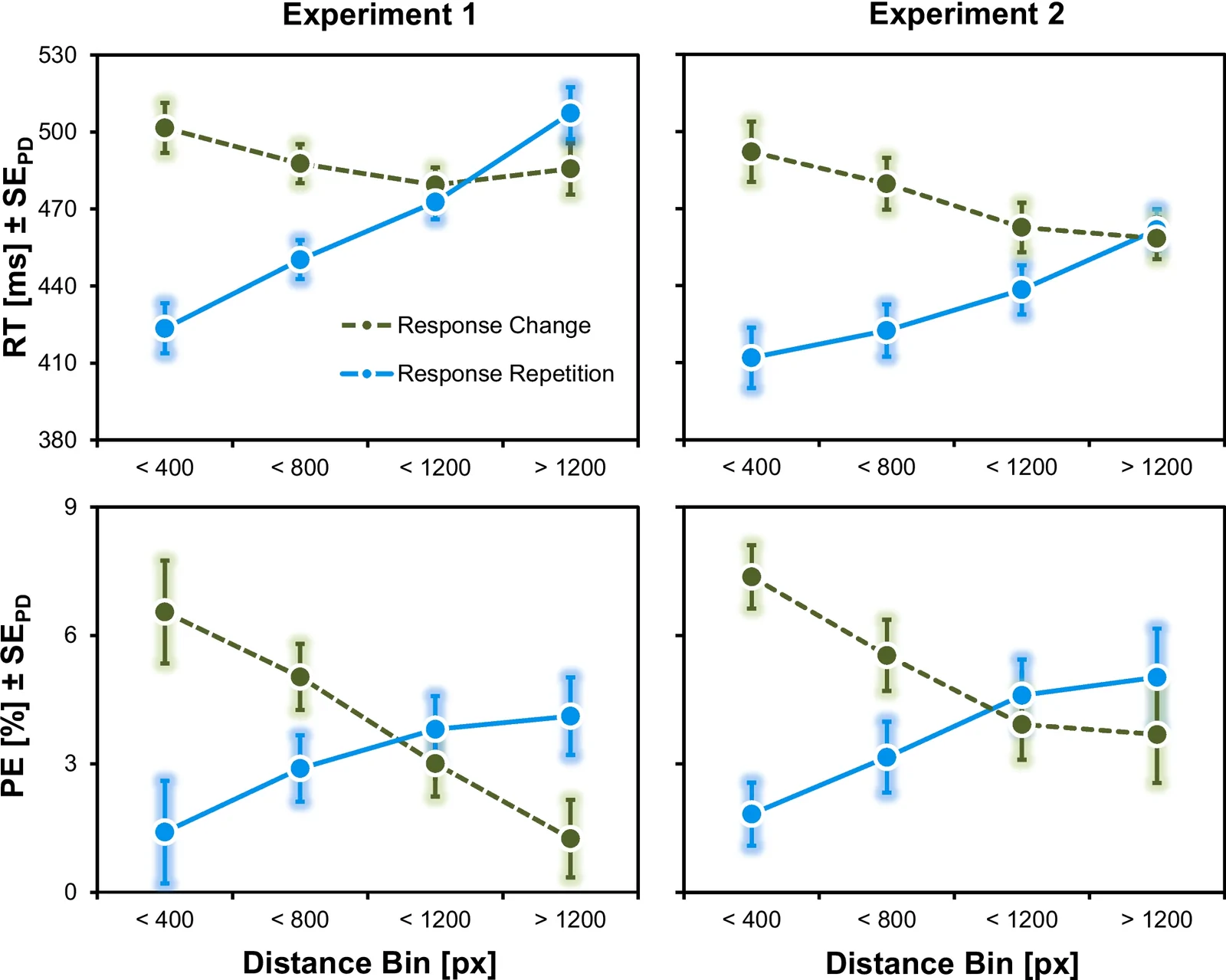
Response time (RT) and error percentage (PE) as a function of response sequence and binned target-target distances across trials. RTs for response repetitions (i.e., trials requiring the same hand as in the preceding trial) increased continuously with distance, whereas RTs for response changes decreased in a similar fashion, indicating continuous rather than categorical representations of either the target position and/or the response location. The same held true for PEs, with response repetitions and changes coding the sequence of correct responses, not the actually executed ones. Error bars indicate standard errors of paired differences (SE_PD_; Pfister & Janczyk, 2013)

This analysis indicated that the added complexity of the continuous model relative to the categorical coding was warranted, with the latter model being ℒ_relative_ <.001 times as probable as the continuous model to minimize the information loss.^1^ Figure 2 supports this conclusion by showing that reaction times increased as a direct function of distance for response repetitions whereas they decreased in a similar fashion for response changes.

To further quantify the continuous representation of target location, we re-assessed the data with regression coefficient analyses (Lorch & Myers, 1990). We thus split the data of each participant between response repetitions and response changes and regressed raw reaction times on target-target distance. For response repetitions, mean reaction times *increased* by 5.95 ms per distance of 100 pixels, 95% CI: [5.04, 6.86] (48 px correspond roughly to 1° visual angle). For response changes, reaction times *decreased* by 2.25 ms per distance of 100 pixels, 95% CI: [1.18, 2.76]. These regression slopes differed significantly from each other, *t*(32) = 16.31, *p* <.001, *d*_*z*_ = 2.84.

#### Error rates

We performed the same model comparisons for error rates as for reaction times. The continuous model suggested a higher error rate on trials that called for response changes than for those that called for response repetitions, *β* = 1.05 (*SE* = 0.11), and increasing error rates with increasing distance, *β* = 1.05 (*SE* = 0.17). These main effects were again qualified by a sizeable interaction, *β* = −1.98 (*SE* = 0.23), and a model comparison to a main effects model indicated the saturated model to be preferable, χ^2^(1) = 76.17, *p* <.001. This interaction was consistent with the pattern observed for reaction times: When responses were to be repeated, participants were more likely to commit an error as the location difference increased, whereas when responses were to be switched, participants were less likely to make an error as location difference increased. This pattern was again better explained by a continuous model than by a categorical model, ℒ_relative_ <.001.

### Discussion

Experiment 1 tested for binding and retrieval of continuous stimulus and response features. Participants pointed to a target using their left or right hand and we manipulated whether stimuli appeared at the same location in two successive trials or whether stimuli changed to a new location. This new location was randomly sampled and the continuous, metric distance of this location to the previous target location affected action planning by promoting response repetitions at small distances and response changes at large distances. Modeling target-target distances across trials as a continuous rather than a categorical predictor better accounted for the data, indicating that binding and retrieval indeed extend to continuous features. In Experiment 2, we aimed to conceptually replicate and extend this finding by manipulating target-target distances in a fully continuous fashion.

## Experiment 2

Whereas in Experiment 1 location repeated on 50% of trials and changed to another random location in the other 50% of trials, in Experiment 2 location was chosen randomly on every trial, with no restrictions other than the stimulus needing to fully fit on the display.

### Methods

#### Open science practices

We report all manipulations, all measures in the study, and all data exclusions. We pre-registered sample size, procedure, and analyses (https://aspredicted.org/gmy5-zdr3.pdf). Anonymized raw data and analysis scripts are available on the Open Science Framework (https://osf.io/f2n4s/).

#### Participants

Thirty-four new participants from the University of Notre Dame participated in exchange for course credit. All participants reported normal or corrected-to-normal visual acuity and color vision. Sample-size considerations were as in Experiment 1, and one dataset was excluded from further analysis due to an insufficient number of valid trials (187/400).

#### Stimuli, apparatus, procedure, and design

Experiment 2 replicated the design of Experiment 1 with the exception that rather than location repeating on 50% of trials, the target appeared at a random location on every trial. Experiment 2 still comprised 400 trials, half of which were left response trials and half of which were right response trials. For analysis, we coded target-target distances across trials and whether trials were response repetitions or changes relative to the previous trial.

### Results

#### Data exclusions

Preprocessing was as in Experiment 1, and we excluded slow responses (0.1%) and screened the data for error trials in which participants failed to hit the target (1.5%) as well as for error trials in which participants responded with the wrong hand (4.45%). A further 2.1% of outlier trials were excluded from the reaction-time analyses.

#### Reaction times

Figure 1B summarizes the main findings of the reaction-time analysis (lower panel). As in Experiment 1, the saturated model yielded longer reaction times for response changes than for response repetitions, *β* = 44.14 (*SE* = 1.93), and increasing reaction times with increasing distance, *β* = 38.57 (*SE* = 3.52). The interaction was again sizeable, *β* = −69.03 (*SE* = 4.94), and a model comparison to a main effects model supported the saturated model including the interaction term, χ^2^(1) = 193.81, *p* <.001.

Even though the design did not implement a distance manipulation that would lend itself to a categorical re-coding, we still opted to compare the continuous model with a cruder categorical coding of distance (small, medium, large; determined by participant-wise tertile splits of the distance distribution). Analyses of relative likelihoods favored the continuous model over a cruder categorical version, ℒ_relative_ <.001, again suggesting that location was represented as a continuous feature.

Regression coefficient analysis indicated that, for response repetitions, mean reaction times increased by 3.90 ms per 100 pixels distance, 95% CI: [3.03, 4.78], whereas for response changes, mean reaction times decreased by 3.07 ms per distance of 100 pixels, 95% CI: [2.06, 4.09]. These mean regression coefficients were significantly different from each other, *t*(32) = 8.78, *p* <.001, *d*_*z*_ = 1.53.

#### Error rates

Error rates were higher on trials that called for response changes compared to response repetitions, *β* = 0.45 (*SE* = 0.09), they increased with increasing distance, *β* = 0.91 (*SE* = 0.18), and this effect was qualified by an interaction, *β* = −1.61 (*SE* = 0.23). A model comparison to a main-effects model favored the saturated model, χ^2^(1) = 49.86, *p* <.001, and a comparison to a categorical coding of distance indicated the continuous model to minimize information loss, ℒ_relative_ =.084.

### Discussion

Experiment 2 replicated and extended the primary findings of Experiment 1 to fully continuous stimulus and response locations. Increasing distances between successive target locations favored response changes over response repetitions whereas decreasing distances favored response repetitions. This lends further support for the idea that binding and retrieval comprise non-categorical, continuous features.

## General discussion

The current study tested whether feature binding and retrieval operate on the level of categorical decision making or whether these processes also incorporate continuous stimulus and response features. We therefore asked participants to perform pointing movements to target stimuli at randomly varying locations. Across both experiments we found that small distances between successive targets facilitated response repetitions whereas large distances facilitated response changes, consistent with episodic binding and retrieval of continuous features.

Binding and retrieval have been invoked as potential mechanisms to explain human behavior in a vast range of settings, including the robust experimental effects of conflict adaptation (Mayr et al., 2003), priming (Henson et al., 2014; Tenpenny, 1995), and task-switch costs (Koch et al., 2018). The current observation of binding and retrieval opens up a new perspective in suggesting that these mechanisms do not only guide efficient decision making; the present findings rather suggest that these mechanisms also operate for non-categorized stimulus features and/or for continuous features of the ensuing movements, thus enabling direct access to the agent’s motor repertoire.

Observing binding and retrieval for continuous features also cannot be explained by competitor models that have been put forward for categorical designs, thus allowing for clean theoretical interpretations. One such alternative view is polarity correspondence (Proctor & Cho, 2006; Proctor & Xiong, 2015). According to the polarity correspondence principle, the cognitive system organizes limited stimulus and response sets along poles such that stimuli and responses artificially become (in)compatible. Because the continuously varying target stimuli of the present experiments cannot be easily re-coded as poles, polarity correspondence cannot easily explain the current data. A second limitation of common categorical designs is that they come with a high probability of joint feature repetitions. This may cause a predictive system to develop biases that, when incorrect, would require correction in case of only partial overlap (e.g., Soetens et al., 1985). By manipulating stimulus and response location continuously we reduced the ability for polarity correspondence or predictive biases to occur, thus yielding clear evidence for binding and retrieval as the source of the observed effects.

A competing view to binding and retrieval has been proposed in terms of the signaling hypothesis (Hazeltine et al., 2024; Koch et al., 2023; Weissman et al., 2023; see also Fletcher & Rabbitt, 1978; Frings et al., 2007; Krueger & Shapiro, 1981). Instead of stimulus repetitions retrieving a bound response as suggested by binding accounts, the signaling hypothesis holds that environmental events serve as decision heuristics for human agents. A stable environment (i.e., a stimulus repetition) signals that no change in behavior is required, thus promoting response repetitions, whereas a changing environment (i.e., stimulus changes) biases the agent to change their response (see also Williams, 1966, for an early account). The present results can be re-interpreted as indicating a similar heuristic that draws on continuous information in that gradual differences between successive situations feed into increasing preparedness to perform distinct responses – despite a general tendency to recycle a previously used motor plan whenever possible (e.g., Jax & Rosenbaum, 2007; Seegelke & Heed, 2025).

Of course, a limitation of our design and the interpretations of the data is that we only manipulated stimulus and response location continuously. Which response hand was required on a given trial still depended on the categorical color feature. We chose to use this method because the interaction between spatially defined stimulus and response features tends to be particularly strong (e.g., Janczyk et al., 2023), and explicit stimulus-response translation has been suggested to be pivotal for observing binding and retrieval effects (Schöpper & Frings, 2024; Schöpper et al., 2022). Future work should therefore aim at extending the current approach to designs that address additional stimulus features (e.g., color of visual stimulation, pitch of auditory stimulation) and response features (e.g., force) to test the limits of binding and retrieval of continuous features. A particularly appealing avenue for such work further involves deciding whether the observed involvement of continuous perceptual features, continuous motor features, or both. The present results suggest such endeavors to be fruitful, especially when making use of spatially and temporally extended reaching movements compared to the discrete keypress responses commonly used in the field.

## Conclusion

Human actions arise from a precise interplay of sensory information and motor activity. The human cognitive system integrates this information into compound representations that allow for retrieving an action plan when re-encountering a situation. Crucially, the present results indicate that such compound representations are not confined to categorical decisions but rather encompass continuous information related to the actual stimulus information and/or motor action.

## Footnotes

1 Potential confounding factors for this analysis are (categorical) side switches across the vertical midline as well as stimulus-response compatibility, for example, in terms of a left stimulus boosting a left response. The crucial interaction of response sequence and distance remained significant when adding these additional predictors to the model, however (see the data and syntax on the Open Science Framework).
